# C-reactive protein-to-albumin ratio improves prediction of 6-month major adverse limb events in diabetic foot ulcers: a retrospective cohort study

**DOI:** 10.3389/fendo.2026.1826193

**Published:** 2026-04-28

**Authors:** Aihemaitijiang Aihetaier, Weilong Qiao, Maiwulangjiang Mohetaer, Maijimi Simayi, Aierpatijiang Aierken, Wulamu Aili, Haobo Cao, Maimaitiyasheng Maimaitituerxun, Shuaijie Liang, Hongwei Yuan, Chengzhi Li

**Affiliations:** Burn and Plastic Surgery, The First People’s Hospital of KaShi, Xinjiang, KaShi, China

**Keywords:** C-reactive protein-to-albumin ratio, diabetic foot ulcer, major adverse limb events, nomogram, risk stratification

## Abstract

**Introduction:**

Diabetic foot ulcers (DFUs) are associated with substantial risks of infection, amputation, and death; however, conventional anatomical classification systems do not fully capture the systemic inflammatory and nutritional status that may influence prognosis. We aimed to evaluate the prognostic value of the C-reactive protein-to-albumin ratio (CAR) and to develop a nomogram for predicting 6-month major adverse limb events (MALE) in hospitalized patients with DFUs.

**Methods:**

We conducted a retrospective cohort study of patients treated at a regional referral center in Northwest China between January 2020 and January 2025. Among 321 screened patients, 139 with complete data were included in the final analysis. MALE was defined as major amputation, unplanned limb revascularization, or death related to foot deterioration or progression of sepsis. Multivariable logistic regression was used to identify independent predictors. Model performance was assessed by discrimination, calibration, bootstrap internal validation, the Brier score, integrated discrimination improvement, net reclassification improvement, and decision curve analysis.

**Results:**

During follow-up, 43 patients (31.0%) experienced MALE. CAR (adjusted odds ratio 1.22, 95% confidence interval 1.03–1.48; P = 0.033) and log-transformed ulcer area (adjusted odds ratio 2.43, 95% confidence interval 1.52–4.10; P < 0.001) were independently associated with MALE. The model including CAR achieved an area under the receiver operating characteristic curve of 0.852, compared with 0.827 for the base model, although the difference was not statistically significant by DeLong testing. Adding CAR improved the Brier score and yielded a positive integrated discrimination improvement. Bootstrap validation showed an optimism-corrected C-index of 0.831, and calibration remained acceptable.

**Discussion:**

CAR was independently associated with 6-month MALE in patients with DFUs. A nomogram incorporating CAR and conventional clinical variables demonstrated good discrimination, calibration, and internal validity, with modest incremental improvement in risk stratification. External validation is warranted before broader clinical application.

## Introduction

Diabetic foot ulcers (DFUs) are among the most serious complications of diabetes mellitus and remain a major cause of infection, hospitalization, lower-extremity amputation, and premature death (1, 2). Contemporary evidence indicates that DFUs affect approximately 18.6 million people worldwide each year and precede most diabetes-related lower-extremity amputations (1). Even after successful healing, recurrence is common, with reported rates of approximately 42% at 1 year and 65% at 5 years (1, 2). In addition, DFUs are associated with substantial long-term mortality, and outcomes are particularly poor after major amputation (3). These observations underscore the need for improved tools to identify high-risk patients at an early stage.

The prognosis of DFUs is determined not only by local wound anatomy but also by infection severity, ischemia, the host inflammatory response, and overall physiological reserve (4). However, traditional classification systems used in routine practice do not fully capture this multidimensional risk. The Wagner classification remains widely used because of its simplicity, but it mainly reflects ulcer depth and the severity of gangrene (5). More recent IWGDF guidance has emphasized that no currently available classification system can be recommended to predict ulcer outcomes in a specific individual, highlighting the continued need for complementary prognostic markers (6). Therefore, biomarkers that reflect systemic disease burden may provide additional prognostic information beyond anatomical staging alone.

The C-reactive protein-to-albumin ratio (CAR) is a simple composite biomarker that integrates inflammatory activity and nutritional reserve. CAR has shown prognostic value in sepsis and other severe inflammatory conditions (7, 8), and recent studies in diabetic foot infection suggest that higher CAR levels are associated with an increased risk of amputation (9). This makes CAR particularly attractive in the setting of DFU, where chronic inflammation, infection, tissue destruction, and malnutrition often coexist and jointly influence healing and limb outcomes.

Nevertheless, the prognostic role of CAR for major adverse limb events (MALE) in hospitalized patients with DFU remains insufficiently defined. Existing prognostic models for diabetic foot outcomes vary substantially in performance and require further refinement and validation before broader clinical use (10). In particular, it remains unclear whether CAR adds meaningful predictive value beyond conventional clinicopathological variables such as ulcer severity, ulcer size, peripheral arterial disease, and infection-related complications.

Accordingly, we conducted a retrospective cohort study to evaluate the association between CAR and 6-month MALE in patients hospitalized with DFU. We further developed and internally validated a nomogram incorporating CAR and conventional predictors, with the aim of providing a pragmatic tool for individualized risk stratification in clinical practice.

## Materials and methods

### Study design and participants

This retrospective cohort study consecutively enrolled patients hospitalized for diabetic foot ulcers (DFUs) in the Department of Burn and Plastic Surgery, The First People’s Hospital of Kashgar Region, between January 2020 and January 2025. The study was conducted in accordance with the Declaration of Helsinki and was approved by the Ethics Committee of The First People’s Hospital of Kashi Prefecture. The requirement for informed consent was waived because of the retrospective study design. Clinical data were extracted from electronic medical records using a standardized data collection form.

### Inclusion and exclusion criteria

Patients were eligible if they met all of the following criteria: (1) age ≥18 years; (2) a diagnosis of type 2 diabetes mellitus according to established diagnostic criteria; (3) a diagnosis of DFU based on the International Working Group on the Diabetic Foot (IWGDF) recommendations (6); and (4) availability of baseline clinical, laboratory, and follow-up data required for outcome assessment and multivariable modeling.

Patients were excluded if they had: (1) major comorbid conditions likely to substantially affect short-term prognosis, including active malignancy, severe hepatic failure, end-stage renal disease, or active autoimmune disease; (2) acute infection outside the foot, major trauma, or major surgery within 4 weeks before admission; or (3) excessive missing data in key candidate variables. The primary analysis was performed as a complete-case analysis. Among 321 screened patients, 139 with complete data were ultimately included.

### Data collection and variable definitions

Baseline variables were collected at admission and included demographic characteristics (age, sex, body mass index [BMI]), medical history (smoking, alcohol use, hypertension, coronary heart disease, and duration of diabetes), and ulcer-related variables. Ulcer characteristics included Wagner grade, ulcer area, peripheral arterial disease (PAD), infection status, and osteomyelitis.

PAD was defined as an ankle-brachial index (ABI) ≤0.90 or imaging evidence of at least 50% stenosis in lower-extremity arteries on vascular ultrasonography or computed tomography angiography. Osteomyelitis was diagnosed according to routine clinical, imaging, and/or microbiological assessments documented in the medical record.

Fasting venous blood samples were collected in the morning after admission. Laboratory measurements included white blood cell count, hemoglobin, fasting blood glucose, glycated hemoglobin (HbA1c), serum albumin, and C-reactive protein (CRP). The C-reactive protein-to-albumin ratio (CAR) was calculated as CRP (mg/L) divided by albumin (g/L).

### Outcome definition

The primary outcome was the occurrence of major adverse limb events (MALE) within 6 months after the index admission. MALE was defined as a composite of: (1) major amputation above the ankle; (2) unplanned limb revascularization, including endovascular or open surgical intervention performed for limb salvage due to disease progression or treatment failure; or (3) death related to foot deterioration or progression of sepsis.

Follow-up data were obtained from outpatient records and telephone interviews. Patients were followed until the first occurrence of a study endpoint or completion of the 6-month follow-up period.

### Statistical analysis

All statistical analyses were performed using R software (version 4.4.0; R Foundation for Statistical Computing, Vienna, Austria). A two-sided P value <0.05 was considered statistically significant.

Continuous variables were assessed for distributional characteristics and are presented as mean ± standard deviation or median (interquartile range), as appropriate. Categorical variables are expressed as counts (percentages). Between-group comparisons were performed using Student’s t test or the Mann-Whitney U test for continuous variables, and the chi-square test or Fisher’s exact test for categorical variables, as appropriate.

To evaluate the association between candidate predictors and MALE, univariable logistic regression analyses were first performed. Based on clinical relevance and the univariable results, the multivariable model included CAR, log-transformed ulcer area, Wagner grade 4, PAD, and osteomyelitis. Although the events per variable (EPV) ratio was approximately 8.6 (43 events for 5 variables)—which is slightly below the traditional rule of 10—variable selection was restricted *a priori* to prevent excessive overfitting, and bootstrap resampling was employed to internally validate the model stability. Odds ratios (ORs) with 95% confidence intervals (CIs) were reported. To assess robustness and facilitate clinical interpretation, additional analyses were performed by modeling CAR as quartiles and as a dichotomous variable using the optimal cutoff derived from the Youden index.

Model discrimination was assessed using the area under the receiver operating characteristic curve (AUC). To evaluate the incremental predictive value of CAR, the AUC of the base model (without CAR) was compared with that of the model including CAR using DeLong’s test for correlated ROC curves. Model calibration was assessed using the Hosmer-Lemeshow goodness-of-fit test, calibration plots generated with bootstrap resampling, and the Brier score.

Internal validation of the final model was performed using bootstrap resampling with 1,000 repetitions. Optimism-corrected performance indices, including the corrected C-index and calibration slope, were derived from the bootstrap procedure. Incremental performance after adding CAR was further assessed using the integrated discrimination improvement (IDI) and continuous net reclassification improvement (NRI), both estimated with bootstrap methods.

A nomogram was constructed based on the final multivariable model using the rms package. Clinical utility was evaluated by decision curve analysis across a range of threshold probabilities. Multicollinearity was examined using variance inflation factors (VIFs), and interaction analyses were performed for CAR with PAD and Wagner grade 4. Key analyses were conducted using the rms, pROC, ResourceSelection, boot, nricens, rmda, car, broom, and ggplot2 packages.

## Results

### Study population and baseline characteristics

**Figure 1:**
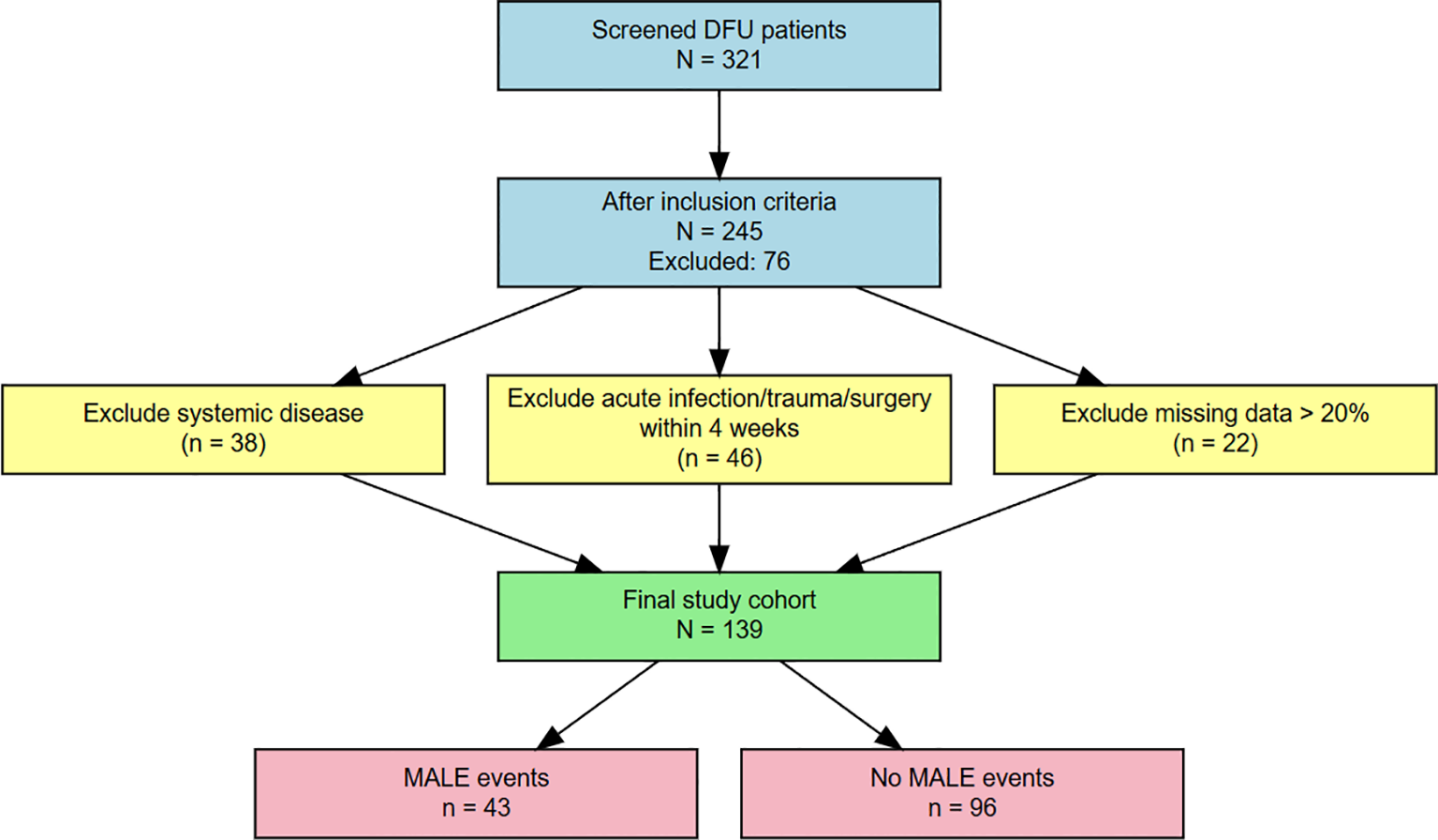
Flowchart of patient selection. A total of 321 hospitalized patients with diabetic foot ulcers were initially screened. After application of the inclusion and exclusion criteria and exclusion of patients with incomplete data, 139 patients were included in the final analysis, of whom 43 experienced major adverse limb events during follow-up and 96 did not.

A total of 321 patients with diabetic foot ulcers (DFUs) were initially screened, and 139 patients with complete data were ultimately included in the present analysis. Among them, 43 patients experienced major adverse limb events (MALEs) during follow-up, whereas 96 did not. The patient selection process is illustrated in **Figure 1**.

Baseline characteristics stratified by MALE status are summarized in **Table 1**. Compared with patients without MALE, those who developed MALE had a longer duration of diabetes (9.12 ± 6.34 vs. 6.69 ± 5.75 years, *P* = 0.011), higher CAR levels (2.89 ± 3.09 vs. 1.03 ± 1.89, *P* < 0.001), and larger ulcer area on the log scale (3.36 ± 1.08 vs. 2.00 ± 1.23, *P* < 0.001). In addition, Wagner grade 4 lesions (48.8% vs. 21.9%, *P* = 0.003) and osteomyelitis (90.7% vs. 51.0%, *P* < 0.001) were more frequent in the MALE group. The prevalence of PAD differed significantly between groups (14.0% vs. 36.5%, *P* = 0.013). No significant between-group differences were observed in age, sex, HbA1c, BMI, insulin use, or ulcer infection.

**Table 1:** Baseline characteristics of patients with diabetic foot ulcers stratified by major adverse limb event status.

Variable	Non_male	Male	P_value
Age	67.74 ± 11.43	68.19 ± 14.62	0.65831
Dm_duration	6.69 ± 5.75	9.12 ± 6.34	0.010681
Hba1c	7.50 ± 2.08	7.74 ± 2.01	0.439722
Bmi	22.94 ± 3.12	23.89 ± 3.87	0.230646
CAR	1.03 ± 1.89	2.89 ± 3.09	3.66E-06
Log_ulcer_area	2.00 ± 1.23	3.36 ± 1.08	2.24E-08
Sex_male	45 (46.9%)	18 (41.9%)	0.715381
Insulin_01	26 (27.1%)	19 (44.2%)	0.072521
Wagner4	21 (21.9%)	21 (48.8%)	0.0027
Pad_dx_01	35 (36.5%)	6 (14%)	0.012841
Osteomyelitis_01	49 (51%)	39 (90.7%)	1.76E-05
Ulcer_infection	76 (79.2%)	40 (93%)	0.074233

### Logistic regression analyses

Univariable and multivariable logistic regression results are presented in **Table 2**. In univariable analysis, CAR was significantly associated with the risk of MALE (OR, 1.37; 95% CI, 1.17–1.66; *P* < 0.001). After adjustment for log-transformed ulcer area, Wagner grade 4, PAD, and osteomyelitis, CAR remained independently associated with MALE (adjusted OR, 1.22; 95% CI, 1.03–1.48; *P* = 0.033). Log-transformed ulcer area was also an independent predictor (adjusted OR, 2.43; 95% CI, 1.52–4.10; *P* < 0.001). In contrast, Wagner grade 4, PAD, and osteomyelitis were not independently associated with MALE in the fully adjusted model.

**Table 2:** Univariable and multivariable logistic regression analyses for 6-month major adverse limb events.

Variable	OR_uni	CI_low_uni	CI_high_uni	P_uni	OR_adj	CI_low_adj	CI_high_adj	P_adj
Age	1.002913	0.974291	1.032992	0.844536				
Dm_duration	0.816	0.390864	1.681429	0.583281				
Hba1c	1.066447	1.005929	1.133071	0.032309				
Bmi	2.13141	1.003029	4.537699	0.048434				
CAR	1.057276	0.886433	1.255483	0.526897				
Log_ulcer_area	1.087873	0.977404	1.214703	0.126384				
Sex_male	1.373594	1.168105	1.658109	0.000355	1.218703	1.026402	1.484416	0.032721
Insulin_01	2.636948	1.842295	3.990698	7.37E-07	2.430892	1.523555	4.104653	0.0004
Wagner4	3.409091	1.58713	7.437705	0.001778	0.536917	0.170134	1.601539	0.273411
Pad_dx_01	0.282625	0.09936	0.694613	0.009692	0.487231	0.146785	1.474357	0.215208
Osteomyelitis_01	9.352041	3.432537	32.97059	7.23E-05	2.384595	0.641365	10.16132	0.208212
Ulcer_infection	3.508772	1.117243	15.52695	0.053176				

**Figure 2:**
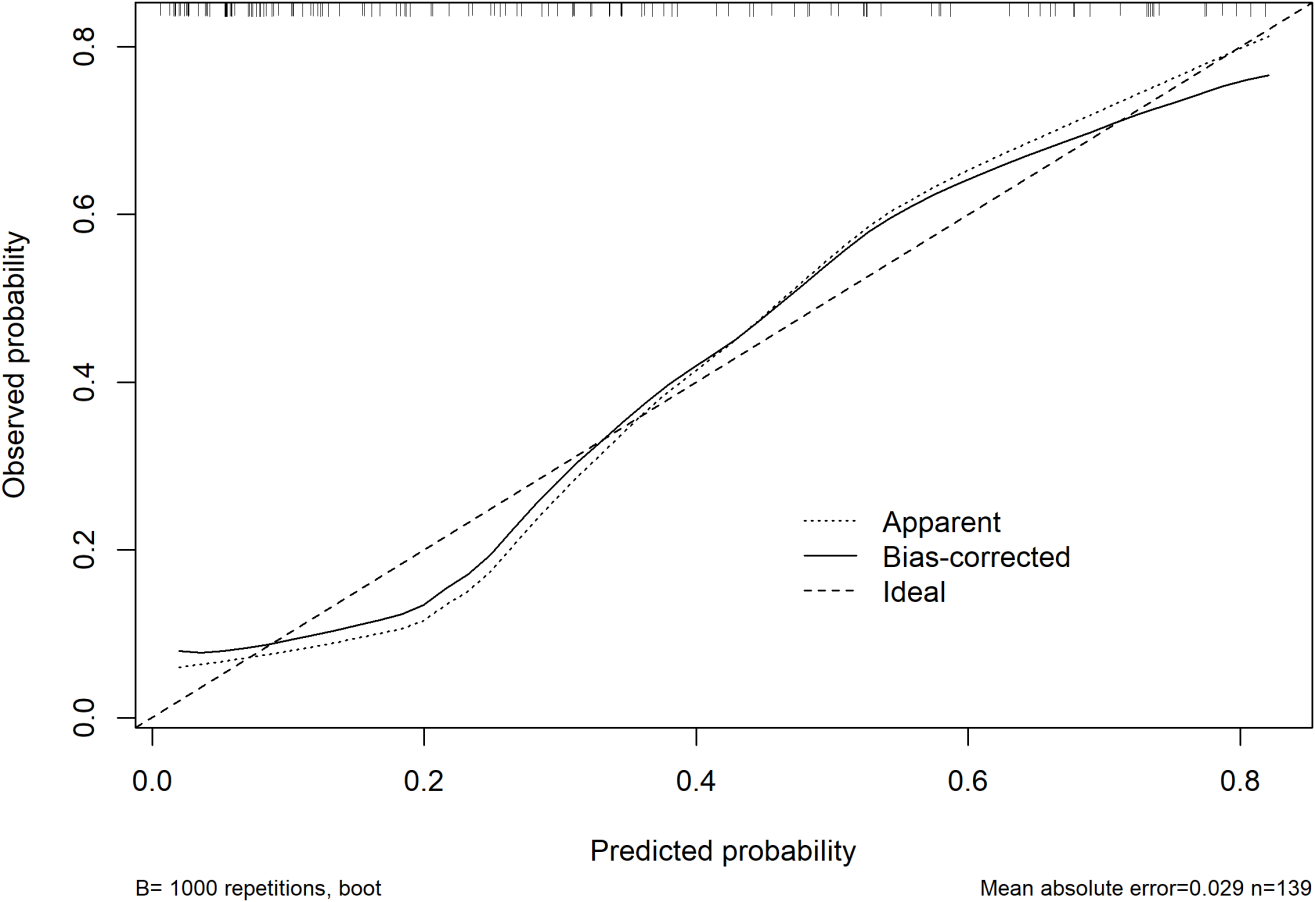
Forest plot of the multivariable logistic regression model for 6-month major adverse limb events. The final multivariable model included the C-reactive protein-to-albumin ratio, log-transformed ulcer area, Wagner grade 4, peripheral arterial disease, and osteomyelitis. Odds ratios are shown with 95% confidence intervals.

A forest plot of the multivariable model is shown in **Figure 2**. Multicollinearity was low, with all variance inflation factors ranging from 1.07 to 1.57. In addition, no significant interaction was observed between CAR and PAD (P for interaction = 0.932) or between CAR and Wagner grade 4 (*P* for interaction = 0.950) (see **Supplementary Table S1**).

### Discrimination, calibration, and incremental model performance

**Figure 3:**
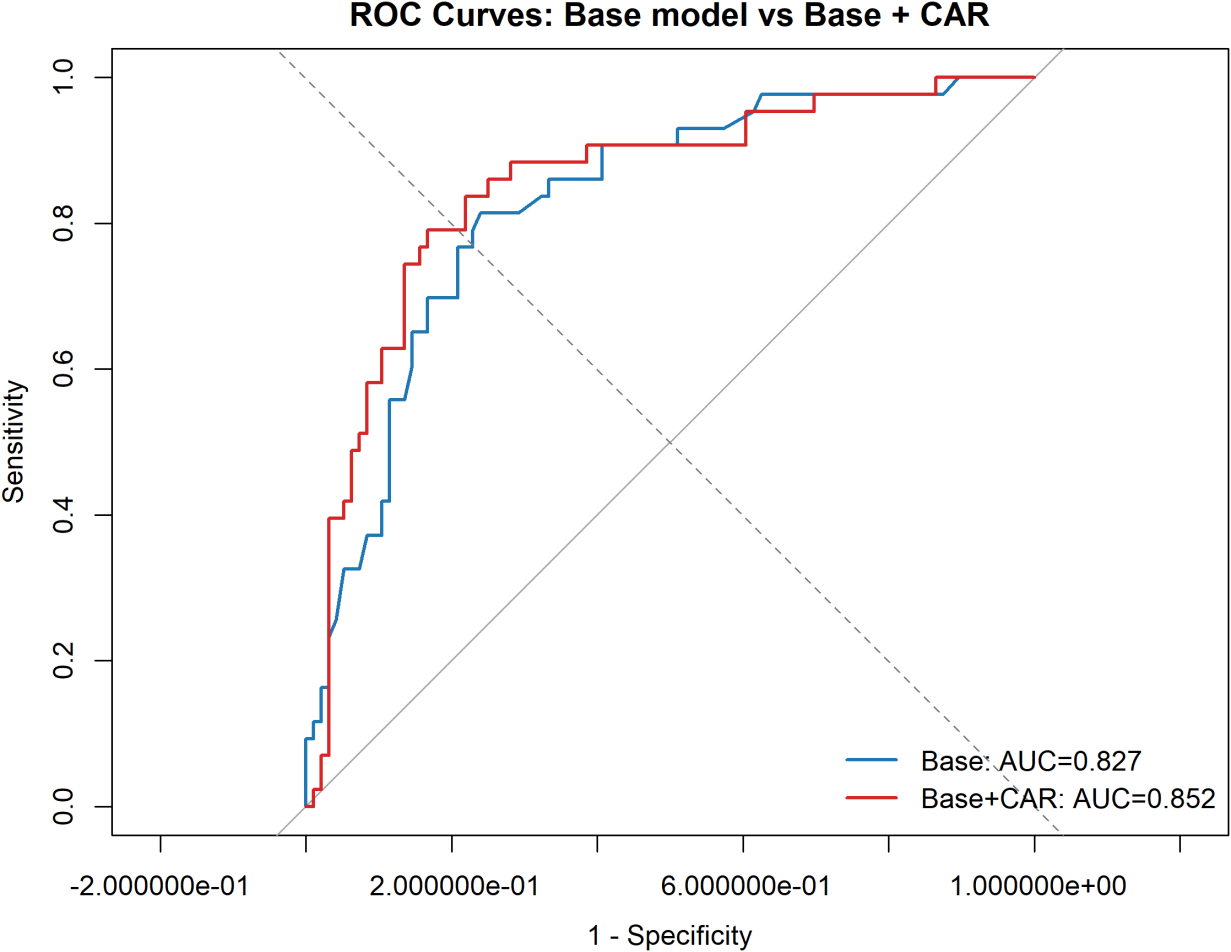
Receiver operating characteristic curves for prediction of 6-month major adverse limb events. The base model included log-transformed ulcer area, Wagner grade 4, peripheral arterial disease, and osteomyelitis. The extended model additionally included the C-reactive protein-to-albumin ratio. The area under the curve was 0.827 for the base model and 0.852 for the extended model.

The discriminatory performance of the models is shown in **Table 3**, **Figure 3**. The base model, which included log-transformed ulcer area, Wagner grade 4, PAD, and osteomyelitis, achieved an AUC of 0.827 (95% CI, 0.754–0.900). After adding CAR, the AUC increased to 0.852 (95% CI, 0.780–0.923). However, the difference between the two correlated ROC curves was not statistically significant by DeLong’s test (*P* = 0.111).

**Table 3:** Discrimination, calibration, and incremental predictive performance of the base model and the model including the C-reactive protein-to-albumin ratio.

Model	AUC	AUC_CI	HL_P	Brier
Base model	0.827156	0.754-0.9	0.547427	0.152685
Base + CAR	0.851502	0.78-0.923	0.244633	0.140424

Model calibration was acceptable for both models, with Hosmer-Lemeshow P values of 0.547 for the base model and 0.245 for the model including CAR. Notably, the Brier score improved from 0.153 in the base model to 0.140 after the inclusion of CAR, indicating better overall prediction accuracy.

To further evaluate the incremental value of CAR, reclassification analyses were performed. The integrated discrimination improvement (IDI) was 0.048, with a bootstrap percentile 95% CI of (0.0004–0.1627) and a BCA 95% CI of (0.0005–0.1729), suggesting a modest but positive improvement in average risk separation after adding CAR. By contrast, the continuous net reclassification improvement (NRI) was 0.222 (95% CI, −0.154 to 0.571), indicating that the overall reclassification gain did not reach statistical significance. Component analysis showed that this change was mainly driven by improved downward reclassification among patients without MALE (NRI− = 0.292; 95% CI, 0.100–0.480), whereas the upward reclassification of patients with MALE was limited (NRI+ = −0.070; 95% CI, −0.373 to 0.228). Detailed reclassification metrics are provided in **Supplementary Table S2**.

### Internal validation and calibration of the final model

**Figure 4:**
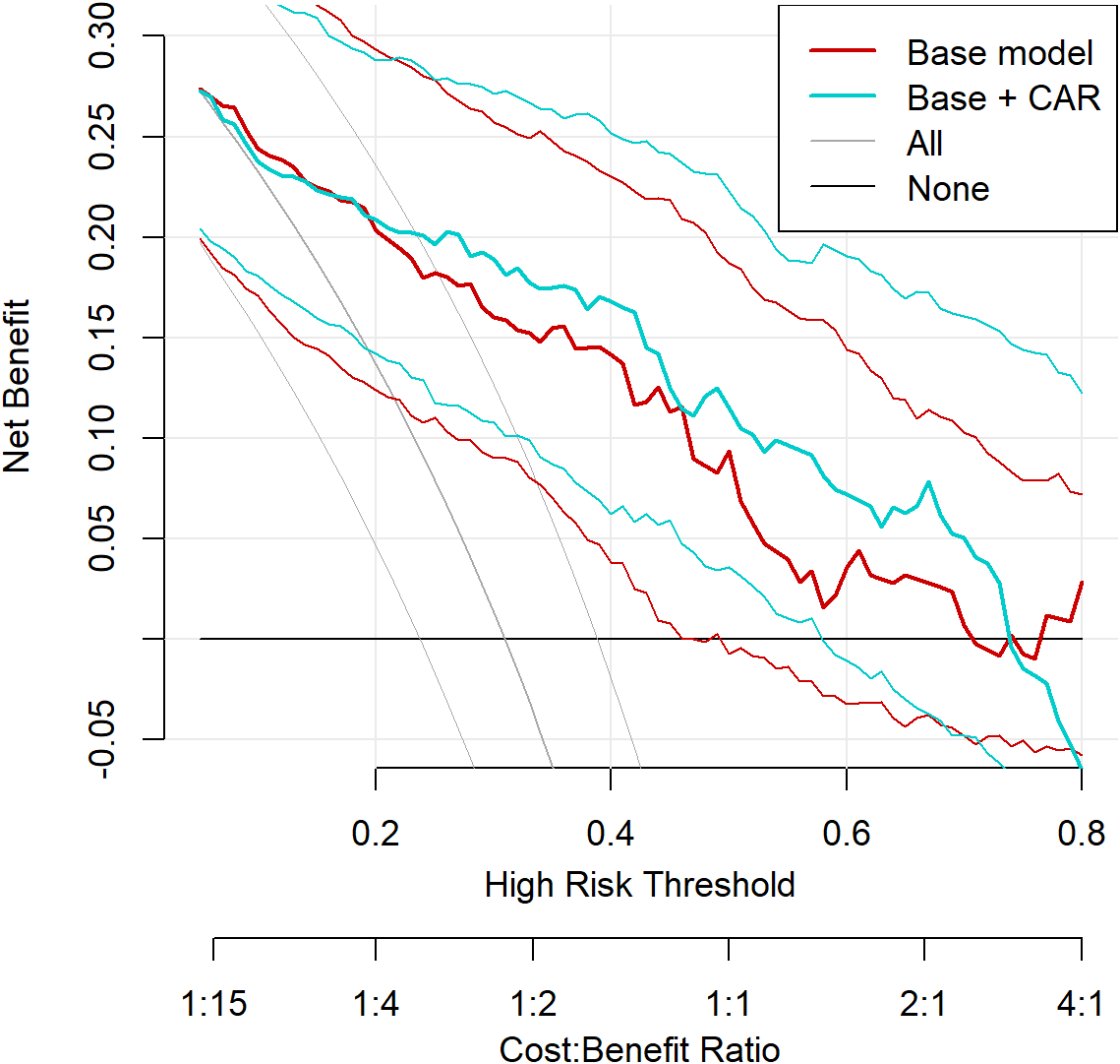
Bootstrap calibration plot of the final prediction model. Calibration of the final model was assessed using 1000 bootstrap resamples. The plot shows the agreement between predicted and observed probabilities of 6-month major adverse limb events.

Bootstrap internal validation of the final model with 1,000 resamples demonstrated good apparent performance with limited optimism. The optimism-corrected Dxy was 0.662, corresponding to an optimism-corrected C-index of approximately 0.831. The optimism-corrected calibration slope was 0.858, and the corrected Brier score was 0.154. The bootstrap calibration curve showed close agreement between predicted and observed probabilities, with a mean absolute error of 0.029 and a mean squared error of 0.00119 (**Figure 4**). Detailed bootstrap validation metrics are shown in **Supplementary Table S3**.

### Additional analyses of CAR: quartiles and clinically applicable cutoff

To further examine the robustness and clinical interpretability of CAR, additional analyses were performed using both quartiles and a binary cutoff. As shown in **Table 4**, when CAR was categorized into quartiles, the highest quartile was independently associated with a markedly increased risk of MALE compared with the lowest quartile (adjusted OR, 12.22; 95% CI, 2.37–96.65; *P* = 0.006), whereas the second and third quartiles did not reach statistical significance.

**Table 4:** Additional analyses of the C-reactive protein-to-albumin ratio using quartiles and a clinically applicable cutoff.

Variable	OR_adj	CI_low	CI_high	P_value
CAR_q2	4.160298	0.823908	31.67125	0.109999
CAR_q3	4.454375	0.86988	34.2324	0.096325
CAR_q4	12.22472	2.37026	96.65097	0.006035

Using ROC analysis of CAR alone, the optimal cutoff value determined by the Youden index was 0.741, yielding a sensitivity of 65.1%, specificity of 71.9%, positive predictive value of 50.9%, and negative predictive value of 82.1%. When CAR was dichotomized according to this cutoff, high CAR remained independently associated with MALE after multivariable adjustment (adjusted OR, 3.61; 95% CI, 1.43–9.50; *P* = 0.008).

### Nomogram construction and decision curve analysis

**Figure 5:**
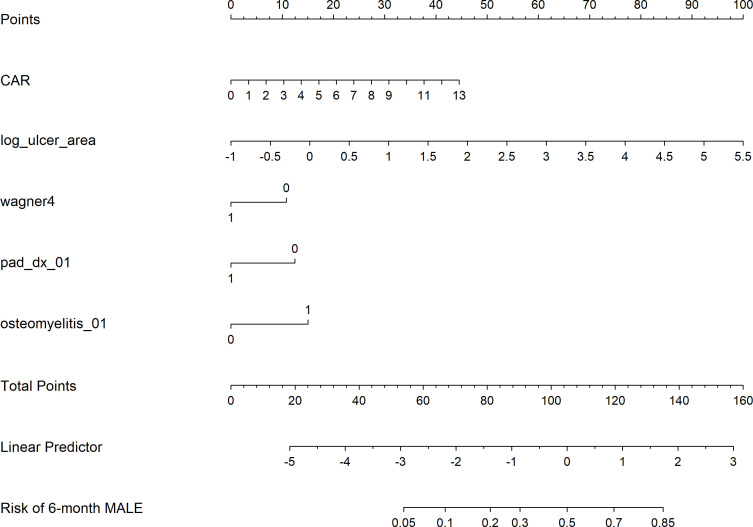
Nomogram for predicting 6-month major adverse limb events in patients with diabetic foot ulcers. The nomogram was developed from the final multivariable model and incorporates the C-reactive protein-to-albumin ratio, log-transformed ulcer area, Wagner grade 4, peripheral arterial disease, and osteomyelitis to estimate the individual probability of 6-month major adverse limb events.

**Figure 6:**
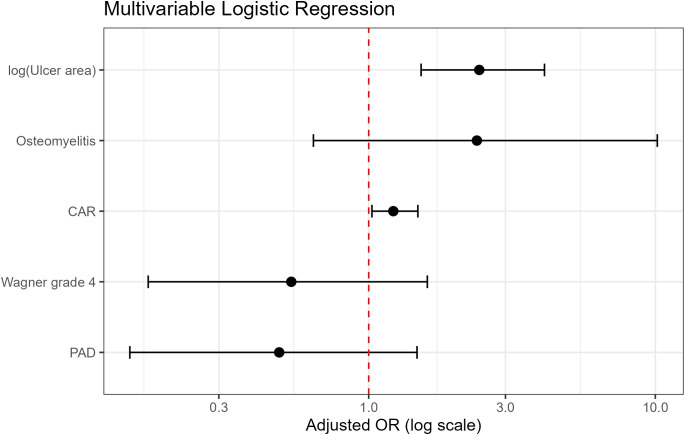
Decision curve analysis of prediction models for 6-month major adverse limb events. Decision curve analysis comparing the base model and the model including the C-reactive protein-to-albumin ratio across clinically relevant threshold probabilities. The model including the biomarker showed greater net clinical benefit across most threshold ranges.

Based on the final multivariable model, a nomogram incorporating CAR, log-transformed ulcer area, Wagner grade 4, PAD, and osteomyelitis was developed to estimate the individual probability of 6-month MALE (**Figure 5**). Decision curve analysis further suggested that the model including CAR provided greater net clinical benefit than the base model alone across most clinically relevant threshold probabilities (**Figure 6**).

## Discussion

DFU remains one of the most devastating complications of diabetes because it is closely linked to infection, amputation, recurrence, and death. Recent high-quality reviews have emphasized that DFUs affect millions of patients worldwide each year, precede most diabetes-related lower-extremity amputations, and continue to impose a major clinical and public health burden. Against this background, there is a persistent need for pragmatic biomarkers that can improve early risk stratification using laboratory data that are inexpensive and routinely available in daily practice (2, 11).

In the present study, we found that CAR was independently associated with 6-month MALE after adjustment for ulcer area, Wagner grade 4, PAD, and osteomyelitis. By explicitly incorporating the Wagner classification—a widely validated and standardized system for grading DFU severity—into our multivariable model. In the present study, CAR remained associated with 6-month MALE after adjustment for ulcer area, Wagner grade 4, PAD, and osteomyelitis. Because Wagner classification is a widely used standardized system for grading DFU severity, its inclusion in the multivariable model helped partially account for baseline ulcer severity. However, residual confounding by ulcer severity cannot be fully excluded in a retrospective study. Therefore, our findings should be interpreted as showing that the association of CAR persisted after adjustment for the available severity-related covariates, rather than proving complete independence from ulcer severity. This association remained evident across alternative analytic strategies: CAR retained significance when dichotomized using the Youden-derived cutoff, and patients in the highest CAR quartile had a substantially higher risk of MALE than those in the lowest quartile. These findings suggest that the relationship between CAR and adverse limb outcomes is relatively robust and not confined to a single modeling approach. Importantly, our results are directionally consistent with emerging studies in diabetic foot infection showing that higher CAR is associated with increased amputation risk, as well as earlier work demonstrating that elevated CRP and reduced albumin are each linked to worse outcomes in diabetic foot disease (10, 12, 13).

The biological plausibility of CAR as a prognostic marker in DFU is strong. CRP reflects systemic inflammatory activation and may mirror infection severity, tissue destruction, and the intensity of the host response. Mechanistically, persistent inflammation in diabetes may contribute to endothelial dysfunction and microvascular impairment, thereby aggravating tissue ischemia and delaying wound healing. In parallel, chronic inflammatory signaling may be associated with immune dysregulation and impaired resolution of inflammation, which can promote ulcer progression and tissue damage (14, 15). Albumin, in contrast, is influenced by both nutritional reserve and inflammatory burden, and hypoalbuminemia has repeatedly been associated with poor surgical and limb-related outcomes (16). A ratio combining these two markers may therefore better capture the complex interplay between systemic inflammation, metabolic exhaustion, and impaired tissue repair than either biomarker alone. This interpretation is also supported by work outside the diabetic foot field, where CAR has shown prognostic value in sepsis and other severe inflammatory states, supporting its broader role as a composite marker of systemic stress (17, 18).

At the same time, our data indicate that the incremental predictive value of CAR should be interpreted with appropriate caution. Although adding CAR increased the AUC from 0.827 to 0.852, this improvement was not statistically significant by DeLong testing (P = 0.111), suggesting a modest tendency toward improved discrimination rather than a dramatic enhancement. In contrast, the IDI was positive, and the Brier score improved after CAR was added, indicating better average separation of predicted risks and better overall prediction accuracy. However, given our relatively small sample size, metrics such as IDI and continuous NRI can be sensitive to model specification and data fluctuations. Therefore, while discrimination and calibration indices support the model’s validity, these reclassification metrics should be viewed as supportive and exploratory. The continuous NRI did not reach statistical significance overall, but the improvement was largely driven by a positive NRI−, suggesting that CAR mainly improved downward reclassification among patients who did not subsequently develop MALE. In practical terms, this pattern implies that CAR may be more useful for refining lower-risk classification than for markedly increasing sensitivity to detect future events. This interpretation also fits the broader prognostic-model literature in diabetic foot, where incremental gains from adding a new biomarker to a model already containing strong clinical predictors are often modest rather than dramatic (9, 19).

Another important finding was that log-transformed ulcer area remained a strong independent predictor in the final model, whereas Wagner grade 4, PAD, and osteomyelitis did not retain independent significance after adjustment. This should not be interpreted as evidence that these traditional factors are clinically unimportant. Rather, it likely reflects overlap among correlated indicators of disease severity in a relatively small cohort. In the broader literature, ulcer severity, PAD, infection severity, and osteomyelitis are consistently associated with limb loss, and current guideline documents continue to regard vascular compromise and infection assessment as central to prognosis and management in diabetic foot disease (20–22). Accordingly, our findings are better viewed as refining the established framework rather than contradicting it.

An unexpected finding in our baseline cohort was the lower prevalence of PAD in the MALE group (14.0%) compared to the non-MALE group (36.5%), alongside a relatively low overall PAD prevalence (29.5%) for a severe DFU population. This counterintuitive observation is likely driven by institutional triage and referral patterns rather than true biological protection. Specifically, patients presenting primarily with severe ischemic symptoms or advanced PAD are typically admitted directly to the vascular surgery department for planned revascularization. In contrast, our cohort was enrolled from the Department of Burn and Plastic Surgery, where patients predominantly present with severe tissue defects, extensive necrosis, or severe infection rather than isolated ischemia. This specific selection bias, combined with the sample size, likely altered the distribution of PAD and attenuated its independent prognostic weight in our multivariable model.

The overall performance of the final model was encouraging. The model including CAR showed good discrimination, acceptable calibration, low apparent calibration error, and limited optimism on bootstrap internal validation, with an optimism-corrected C-index of approximately 0.83. From a modeling perspective, this level of performance is clinically respectable in DFU prognostic research, where model discrimination often varies considerably across derivation and validation cohorts. In addition, the decision curve analysis suggested that the model including CAR may provide greater net clinical benefit than the base model across a range of clinically relevant threshold probabilities. Together, these findings support CAR as a useful adjunctive marker in bedside risk stratification rather than a stand-alone discriminator.

The dichotomized analysis further improves the clinical interpretability of CAR. Using a data-driven cutoff of 0.741, high CAR was associated with more than a threefold increase in the adjusted odds of MALE. Although any threshold derived from a single-center cohort should be interpreted cautiously before external validation, this result suggests that CAR may be useful not only as a continuous modeling variable but also as a clinically accessible marker to identify patients who warrant closer surveillance, more intensive infection control, and earlier multidisciplinary intervention. This potential bedside utility is attractive because CRP and albumin are routinely measured in many patients hospitalized for diabetic foot disease (23–25).

Several limitations should be acknowledged. First, this was a retrospective single-center study with a relatively small sample size. With 43 events and 5 predictor variables, our multivariable model yielded an EPV ratio of approximately 8.6. While slightly below the conventional threshold of 10, we mitigated the risk of overfitting by strictly limiting candidate variables and utilizing bootstrap internal validation. Nevertheless, this limited sample size increases the risk of overfitting and may partly explain why some established predictors did not remain independently significant in the fully adjusted model. Second, only internal validation was performed; therefore, the generalizability of the model and the proposed CAR cutoff remains uncertain. Because patient baseline characteristics, wound care protocols, and revascularization practices can vary substantially across different institutions, external validation in independent, multicenter cohorts is strictly required before this predictive model can be confidently applied in broader clinical settings. Furthermore, due to the retrospective design, we could not systematically capture granular details regarding highly heterogeneous treatment strategies, such as specific antibiotic regimens or diverse wound care practices. Because these therapeutic interventions independently influence inflammatory trajectories and clinical outcomes, their absence introduces potential residual confounding. Third, CAR was assessed at baseline only. Because a patient’s inflammatory and nutritional status can fluctuate dynamically in response to infection progression or treatment, a single measurement limits its utility as a real-time monitoring tool. We could not determine whether serial, longitudinal changes in CAR during treatment would improve prognostic accuracy, which remains a key area for future investigation. Fourth, the use of a composite MALE endpoint may combine events with partially different biological mechanisms and clinical decision pathways. Specifically, revascularization can be heavily influenced by anatomical considerations, institutional practice patterns, and operator preferences, making it conceptually distinct from definitive patient-centered outcomes like major amputation or death. Future studies should consider analyzing amputation-free survival (a composite of major amputation and death) as the primary outcome, with revascularization evaluated separately as a secondary endpoint. Finally, although the IDI and Brier score supported incremental value, the non-significant DeLong test and overall NRI indicate that the added value of CAR is moderate rather than transformative. These issues should be explicitly acknowledged to keep the interpretation appropriately balanced. Furthermore, while this study focused strictly on limb-threatening events, time-to-ulcer healing is a critical patient-centered outcome. Unfortunately, systematic and precise data on wound closure times were not fully available for this retrospective cohort; exploring the association between CAR and ulcer healing trajectories remains an important avenue for future research.

Future studies should focus on external validation in larger multicenter cohorts, direct comparison of CAR with other inflammatory or immunonutritional indices, and evaluation of whether repeated CAR measurements improve temporal prediction of limb-related outcomes. It would also be valuable to assess whether CAR performs differently across specific subgroups, such as patients with severe infection, advanced ischemia, renal dysfunction, or osteomyelitis. In addition, separating major amputation, revascularization, and death in sufficiently powered cohorts may clarify whether CAR is more strongly related to local limb threat, systemic inflammatory burden, or both.

## Conclusion

In conclusion, our study suggests that CAR is a clinically accessible biomarker associated with 6-month MALE in patients with DFU. When added to a model containing ulcer area and other conventional predictors, CAR provided modest incremental improvement in risk stratification, particularly in identifying patients less likely to experience adverse limb events. These findings support the potential role of CAR in pragmatic prognostic assessment, while underscoring the need for external validation before broader clinical application.

## Data Availability

The raw data supporting the conclusions of this article will be made available by the authors, without undue reservation.

## Supplementary material

Supplementary Table 1 Collinearity diagnostics and interaction tests for the final multivariable model. Panel **(A)** Variance inflation factors for the variables included in the final model. Panel **(B)** Interaction tests for the C-reactive protein-to-albumin ratio with peripheral arterial disease and Wagner grade 4.

Supplementary Table 2 Detailed reclassification metrics after adding the C-reactive protein-to-albumin ratio to the base model.

Supplementary Table 3 Detailed bootstrap internal validation and calibration metrics of the final model.
